# Nostalgia, Gratitude, or Optimism: The Impact of a Two-Week Intervention on Well-Being During COVID-19

**DOI:** 10.1007/s10902-022-00513-6

**Published:** 2022-03-19

**Authors:** Amelia Dennis, Jane Ogden

**Affiliations:** School of Psychology, University of Surrey, Guildford, UK

**Keywords:** COVID-19, Nostalgia, Gratitude, Best possible self, Well-being

## Abstract

Research indicates that brief 2-min positive psychology interventions (PPIs) increase well-being during COVID-19 lockdowns. The present study extended this to assess the effectiveness over two-weeks. Participants (*n* = 150) were randomly allocated to one of three PPIs; nostalgia, gratitude, best possible self (BPS), or control. The interventions were slightly adapted for the lockdown and were completed three times, every seven days over two-weeks. Well-being measures were completed immediately after the first intervention (T1), after the next two interventions (T2−T3) and at one-week follow-up (T4) (but no baseline measure of well-being was taken). At T1, participants in the nostalgia, gratitude, and BPS intervention had higher self-esteem than those in the control intervention. At T1 and T2, participants in the gratitude and BPS intervention reported higher social connectedness than participants in the nostalgia and control intervention. Then at follow-up (T4), participants in the nostalgia, gratitude, and BPS intervention had lower fear of COVID-19 than those in the control intervention. Overall, the results show the benefits of nostalgia, gratitude and optimism, compared to the control, during lockdown.

**Supplementary Information:**

The online version contains supplementary material available at 10.1007/s10902-022-00513-6.

## Introduction

As the novel coronavirus (COVID-19) spread around the world, many countries implemented lockdown and stay at home orders. In a bid to control the spread of COVID-19, the United Kingdom (UK) went into a lockdown on the 23rd March 2020 that was lifted at the end of May. Then as COVID-19 continued to spread, England, Wales, Scotland, and Northern Ireland all implemented additional lockdowns in November 2020 (BBC, 2020a, 2020b; Welsh Government, 2020; Government UK, 2020).

The COVID-19 pandemic and resulting lockdowns have had negative consequences on mental health. Research to date has identified reduced well-being and increased anxiety, depression, fear, and stress due to COVID-19 (O’Connor et al., 2020; Torales et al., 2020). Given the role of social interaction in supporting well-being, it is not surprising that during the first UK lockdown, there was a high prevalence of loneliness (Groarke et al., 2020). In addition, COVID-19 has also brought about fear of COVID-19 and COVID-19 health anxiety (the fear or apprehension of catching or having COVID-19). Fear of COVID-19 impedes well-being and quality of life while increasing depression (Alyami et al., 2021; Mahmud et al., 2021). The negative impact of lockdown on well-being has been shown to continue even with the easing of restrictions (Pieh et al., 2021). Additionally, longitudinal studies of previous infection outbreaks show high levels of mental health illness persist long-term (Mak et al., 2009; Reynolds et al., 2008). Due to the potential for the negative psychological impact of COVID-19 to be long-lasting, it is key to identify potentially longer-term solutions to increase well-being during and after lockdowns. One way to increase well-being during the COVID-19 lockdowns is through positive psychology interventions (PPIs). PPIs aim to induce positive emotion, behaviour, or cognition to increase well-being (Sin & Lyubomirsky, 2009).

During the first UK lockdown, Dennis et al. (2020) compared the effectiveness of two minute online positive psychology interventions with different time-orientations: nostalgia focusing on the past, gratitude focusing on the present, and best possible self (BPS) focusing on the future. Dennis et al. (2020) found that BPS and gratitude interventions were more effective at increasing immediate feelings of social connectedness compared to the nostalgia intervention. In addition, the BPS intervention was better at increasing positive affect (PA) compared to the nostalgia intervention. These results suggest that PPIs focusing on the present and future (gratitude and optimism) may increase well-being better than past focused PPIs (nostalgia) during the COVID-19 pandemic and times of uncertainty. Other research has also compared the time orientation of PPIs. Wellenzohn et al. (2016) assessed a past, present, or future time focus of three ‘funny things’, finding that all three time foci increased happiness and reduced depressive symptoms, but the past focus was better at increasing happiness and present superior was better at reducing depressive symptoms. Additionally, Carrillo et al. (2021) compared the effectiveness of individuals’ writing about their best past self, best present self, or best future self. All three interventions increased well-being compared to the control but there were no differences between the three interventions, suggesting that time-orientation does not always influence effectiveness.

The current study will compare three PPIs, each with different time-orientations: nostalgia (past), gratitude (present), and BPS (future), at increasing well-being and reducing fear of catching COVID-19, over two-weeks. By doing this we will attempt to extend and replicate the findings by Dennis et al. (2020).

### Nostalgia

Nostalgia is a positive emotion (Van Tilburg et al., 2019) defined as a ‘sentimental longing or wistful affection for the past’ (The New Oxford Dictionary of English, 1998, p. 1266). Previous research has shown that the nostalgia intervention, compared to a control, increases well-being related constructs such as social connectedness (Abakoumkin et al., 2019), self-continuity (Hong et al., 2021), self-esteem (Cheung et al., 2013; Cheung et al., 2016), and meaning (Sedikides & Wildschut, 2018). In addition, nostalgia interventions also hold the ability to buffer against psychological threats, such as death awareness (Routledge et al., 2008), meaninglessness (Routledge et al., 2011), and self-discontinuity (Sedikides et al., 2015a). Additionally, it is theoretically likely that nostalgia can decrease fear of COVID-19, first, due to its ability to buffer negative psychological states and maintain psychological homeostasis (Sedikides et al., 2015b) and second, due to nostalgia’s ability to increase subjective vitality (feelings of aliveness and energy; Sedikides et al., 2016) and health optimism (Kersten et al., 2016). Therefore, nostalgia is a past focused psychological resource that can bolster well-being and counter negative states (Sedikides et al., 2015b).

### Gratitude

Gratitude is ‘a sense of thankfulness and joy in response to receiving a gift, whether the gift be a tangible benefit from a specific other or a moment of peaceful bliss evoked by natural beauty’ (Emmons, 2004, p. 554) and is, therefore, present focused. Research, including meta-analyses, have shown that gratitude interventions increase well-being, happiness, life satisfaction, self-esteem, and PA (Davis et al., 2016; Dickens, 2017; Rash et al., 2011). Gratitude has also been shown to mitigate distress, with gratitude interventions reducing the symptoms of anxiety, depression, and aspects of health anxiety (e.g., death worry) (Cregg & Cheavens, 2021; Otto et al., 2016). Thus, the gratitude intervention may mitigate the fear of COVID-19, as well as increase well-being.

### Best Possible Self

BPS is an intervention based on inducing optimism whereby participants project themselves into the future and imagine everything has turned out as well as it could (Peters et al., 2010). Meta-analyses have indicated that the BPS intervention increases PA, well-being, and optimism, compared to a control (Carrillo et al., 2019a; Malouff & Schutte, 2017). Additionally, a recent meta-analysis highlights the momentary increases in PA and positive future expectations from BPS compared to a control (Heekerens & Eid, 2021). The BPS intervention has also been shown to reduce symptoms of mental illness, for example, reducing negative affect (NA) that in turn reduces depressive symptoms and increases life satisfaction (Liau et al., 2016). There is also reason to expect BPS to reduce fear of COVID-19 as people with high optimism take action to reduce their health risk, increasing confidence that their efforts will be successful (Carver et al., 2010). In sum, the BPS intervention is a future orientated approach that may increase well-being and reduce fear of COVID-19.

### The Current Study

To date, some research has assessed the repeated effects of BPS and gratitude interventions. Most of this research shows the efficacy of BPS, with the BPS intervention, but not the gratitude intervention, increasing life satisfaction and self-esteem (Owens & Patterson, 2013; Peters et al., 2013). Similarly, a meta-analysis (Carrillo et al., 2019a) found the BPS intervention was more effective at increasing PA and reducing NA than the gratitude intervention. Whereas, Sheldon and Lyubomirsky (2006) found that the repeated gratitude or BPS increased PA and decreased NA. One study has assessed the effect of repeating the nostalgia intervention (Layous et al., 2021), finding that three weeks of engaging in the nostalgia intervention led to greater well-being than the control intervention. These results, taken with the above research comparing BPS and gratitude interventions, may suggest that the BPS intervention will be the most effective; however, no studies have assessed the effects of PPI on fear of COVID-19 or compared repeated use of nostalgia as a result we did not make specific hypotheses.

Only one study (Dennis et al., 2020) compared the effectiveness of the three PPIs (nostalgia, gratitude, and BPS) and reported that both BPS and gratitude interventions increased well-being more than the nostalgia intervention. However, this study only looked at the immediate effects of PPIs rather than the impact of repeated engagement with PPIs. Thus, we aimed to replicate and extend Dennis et al.’s (2020) intervention and conducted three weekly interventions (T1-T3) with a one-week follow-up (T4) that overlapped with UK lockdown restrictions. In the current study, we also extended the outcomes and assessed the effect of PPIs on fear of COVID-19 and well-being due to the negative impact of fear of COVID-19. Therefore, the first purpose of this study was to replicate Dennis et al. (2020) by comparing the immediate effects of each intervention and follow-up on well-being and fear of COVID-19. The second purpose of this study was to assess the effect of the intervention on well-being and fear of COVID-19 over the course of the intervention, to extend Dennis et al. (2020). In assessing the longer-term effects of PPIs, we can first identify the most effective intervention and second identify the effectiveness of repeated engagement of these interventions during lockdowns.

## Method

### Design

The current study used an experimental design. Participants were randomised to one of three interventions: nostalgia, gratitude, BPS, or a control. Each intervention took two minutes and was repeated three times at seven-day intervals. Demographic and self-care behaviour measures were taken before the first intervention. Well-being and fear of COVID-19 measures were taken immediately after the first intervention for T1, then after the second intervention for T2 and the third intervention for T3. Follow-up measures of well-being were taken a week later (T4).

We did not conduct a baseline measure. This was due to considerations of questionnaire fatigue and priming effects for the participant. Further, randomisation through the third party of Qualtrics together with the control condition should control for any third-factor variables and differences between conditions prior to the intervention.

### Participants

**Fig. 1:**
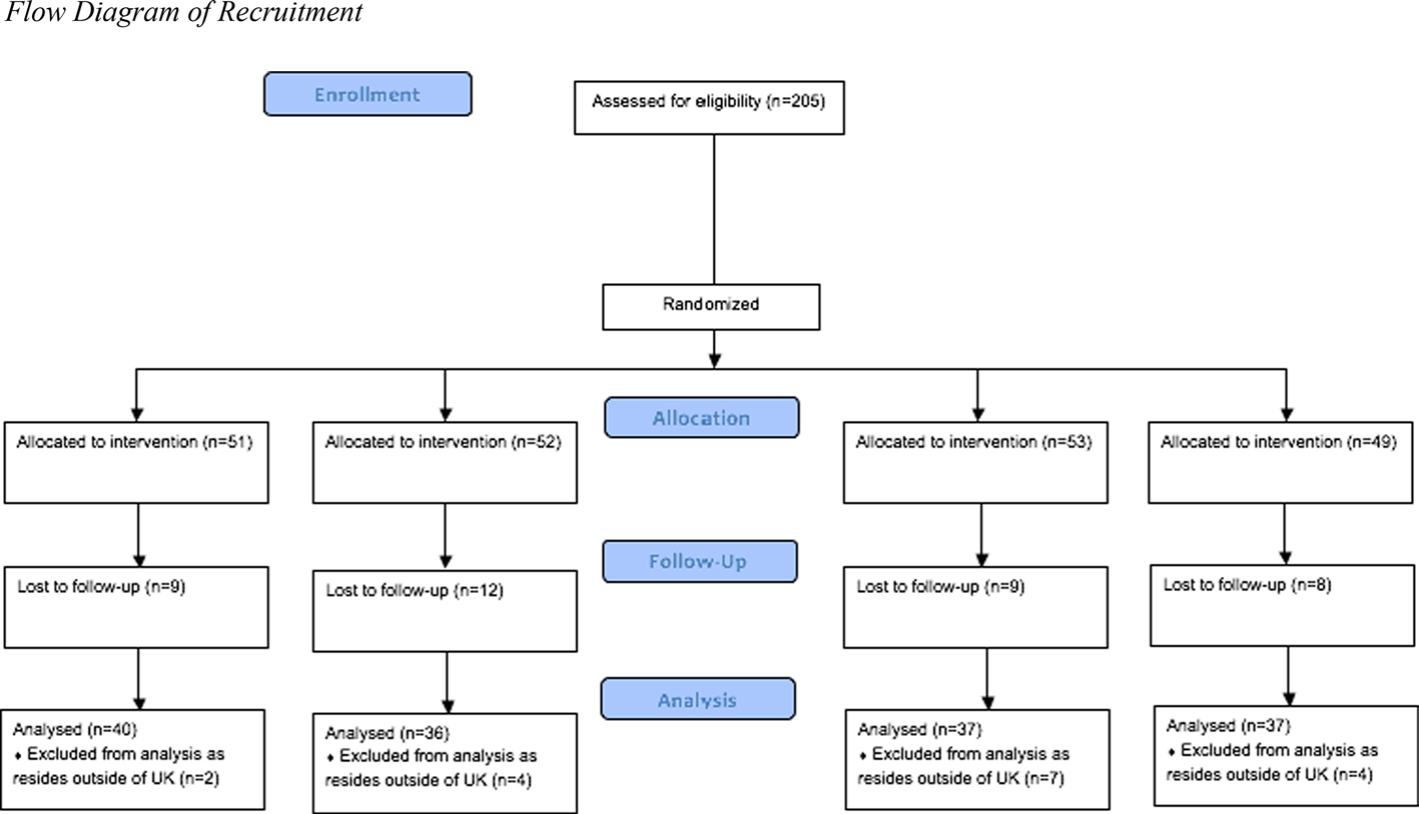
Flow diagram of recruitment

The final sample comprised 150 participants, who all completed T1-T4 and resided in the UK, see Fig. 1 for a flowchart of dropouts. A priori power calculation (G*Power; Faul et al., 2007) indicated a required sample size of 143 based on an ANCOVA with η_p_ ^2^ = 0.073 (Peters et al., 2010), power at 0.8, alpha at 0.05, four groups, and three covariates. We achieved the target sample size. Participants were recruited through university in exchange for lab tokens (*n* = 80), an online survey participant base (Prolific.ac) with monetary compensation (*n* = 45), or opportunity sampling (*n* = 25).

### Interventions

Participants were randomly allocated by Qualtrics, blinded to the researchers, to one intervention at T1 and received the same intervention at T2 and T3. In all interventions, participants had to stay on the intervention page for two minutes. They were also prompted to write their nostalgic experience (nostalgia intervention), three things they are grateful for and why (gratitude intervention), their best possible self (BPS intervention), or a summary of the tv plot (control).

#### Nostalgia

Participants were induced in nostalgia through an adapted Event Reflection Task (ERT; Sedikides et al., 2015b). Participants were given the definition of nostalgia (*‘sentimental longing for the past’) and asked to ‘*think of **a nostalgic event** in your life that occurred before the lockdown. Specifically, try to think of a past event that makes you feel most nostalgic.’ Then, at T2 and T3, participants were additionally told, ‘You can either think and write about the same event as last week or choose a new one.’

#### Gratitude

Participants in the gratitude condition completed Three Good Things (TGT; Seligman et al., 2005) and were asked to ‘think of **three things that you are grateful for today.’ No changes to the intervention were made at T2 or T3.**

#### Best Possible Self

Participants in the BPS condition completed an adapted BPS intervention (Peters et al., 2010). Participants were instructed to ‘imagine yourself in the future, after the lockdown has been lifted and after everything has gone as well as it possibly could. **‘At T2 and T3, participants were also told,’**You can either think and write about the same aspect of your best possible self as last week or choose a new one (e.g., personal, professional).’

### Control

As in Dennis et al. (2020), participants in the control condition were asked to recall a recently watched television plot. Participants were asked to ‘bring to mind a **plot of a show** that you watched recently.’ At T2 and T3, participants were also told ‘You can either think and write about the same tv plot as last week or choose a new one.’

### Measures

All descriptive statistics and reliability estimates for T1-T4 are reported in Table 1.

**Table 1:** Descriptive statistics and internal consistency estimates

	T1			T2			T3			T4		
	M	SD	α	M	SD	α	M	SD	α	M	SD	α
State Nostalgia	3.98	1.42	.97	4.00	1.49	.97	3.86	1.52	.98			
State Gratitude	4.58	1.12	.89	4.69	1.03	.87	4.73	0.98	.84			
State Optimism	4.33	1.08	.86	4.36	1.05	.87	4.34	1.07	.84			
Social Connectedness	4.76	0.91	.87	4.71	1.02	.89	4.64	1.08	.91	4.72	1.00	.91
Self-Esteem	4.20	1.21	.94	4.23	1.17	.94	4.32	1.16	.94	4.31	1.16	.95
Optimism	4.37	1.03	.87	4.33	1.12	.90	4.34	1.16	.93	4.32	1.11	.92
Meaning	4.74	1.03	.90	4.68	1.08	.93	4.63	1.15	.92	4.58	1.13	.93
Happiness	4.39	1.29	.88	4.45	1.27	.89	4.52	1.19	.87	4.45	1.29	.91
Fear of COVID-19	2.20	0.82	.88	2.07	0.85	.89	1.98	0.92	.92	1.92	0.91	.92

### Lockdown behaviour

**Lockdown Characteristics.** Participants described their lockdown lifestyle through five questions in terms of the following: i) the number of people they lived with ii) their lockdown behaviours in terms of how many times in the last two weeks they had left the house, left the house for work, left the house for exercise, and socially interacted (virtually or in-person) with people they did not live with on scales from 1 (Not at all) to 4 (More than once a day).

**Self-Care.** We assessed self-care through four sub-scales of the Mindful Self Care Scale (Cook-Cottone & Guyker, 2018; α = 0.84). The four sub-scaled used were physical care, supportive relationships, self-compassion and purpose, and supportive structure. Participants were asked to rate how frequently they had engaged in each behaviour in the last seven days from 1 (Never: 0 days) to 5 (Regularly: 6–7 days).

### Outcome Measures

**Well-Being.** Participants completed measures of optimism (Cheung et al., 2013; α = 0.87—0.93), meaning (Hepper et al., 2012; Routledge et al., 2011; α = 0.90—0.93), self-esteem (Hepper et al., 2012; α = 0.94—0.95), and social connectedness (Cheung et al., 2013; α = 0.87—0.91). All three sub-scales included four questions, all preceded by ‘Right now, I am feeling…’ and were rated on a scale from 1 (Strongly Disagree) to 6 (Strongly Agree).

We also assessed happiness through the Subjective Happiness Scale (Lyubomirsky & Lepper, 1999; α = 0.90—0.93), a four-item scale to measure happiness through items such as ‘Some people are generally very happy. They enjoy life regardless of what is going on, getting the most out of everything. To what extent does this characterisation describe you?’ Participants rated each question on a scale from 1 to 4.

**Fear of COVID-19.** Participants completed the Fear of Coronavirus-19 scale (Ahorsu et al., 2020; α = 0.88—0.92), a 7-item measure that captures the fear of COVID-19, e.g., ‘I am most afraid of COVID-19.’ All items were preceded by ‘Right now…’ and were rated on a scale from 1 (Strongly Disagree) to 5 (Strongly Agree).

These measures have been used previously and have been shown to have good internal reliability (Cheung et al., 2016; Cook-Cottone & Guyker, 2018; Lyubomirsky & Lepper, 1999; Reznik et al., 2021; Sedikidies et al., 2018).

### Manipulation Checks

We assessed the effectiveness of interventions at inducing nostalgia, gratitude, or optimism through manipulation checks. All questions were preceded by the stem ‘**When I brought to mind my image…’** and measured on a scale from 1 (Strongly Disagree) to 6 (Strongly Agree). The Nostalgia Manipulation Check (Wildschut et al., 2006; α = 0.97—0.98) was used to measure state nostalgia through three items: ‘I had nostalgic feelings’, ‘I was feeling quite nostalgic’, and ‘I felt nostalgic’. An adapted version of gratitude-related feelings (Emmons & McCollough, 2003; α = 0.84—0.89), three-items were used to measure state gratitude: ‘I felt grateful’, ‘I felt grateful for today’, and ‘I felt thankful.’ An adapted version of the dispositional Life Orientation Test-Revised (Scheier et al., 1994; α = 0.84—0.87) was used to measure state optimism through three items: ‘I thought good things will happen to me’, ‘I thought things will turn out as I hoped’, and ‘I was optimist for the future’.

### Procedure

Participants were all recruited between 5 and 20th November 2020 (T1), and data collection of T2-T4 extended until 13th December 2020. The recruitment and data collection of this study coincided with the second UK lockdown. Participants completed the study online through Qualtrics. Before the first intervention, participants completed measures of demographic, lockdown characteristics, and self-care behaviours. They then completed the intervention and immediately after answered measures of well-being, fear of COVID-19, and manipulation checks for all conditions (T1). After seven- and fourteen-days, participants received an email, with a link to the study, at 7AM to complete the following two interventions (T2–T3) followed by measures of well-being, fear of COVID-19, and manipulation checks for all conditions. Finally, one week later, participants completed follow-up measures of well-being and fear of COVID-19 (T4).

## Results

### Data Analysis

We first assessed participants demographics for the whole sample and then by intervention using descriptive statistics, ANOVA, and X^2^. Then we conducted manipulation checks for state nostalgia, gratitude, and optimism T1-T3. Next, we assessed the outcomes of the interventions through ANCOVA and post hoc tests with Bonferroni correction, separately for each time-point (T1-T4). Last, we conducted a 4 (time: T1-T4) × 4 (intervention: nostalgia, gratitude, BPS, control) mixed ANCOVA to assess the effect of the intervention on well-being over time. A Pearson’s correlation was conducted to assess associations between all variables and is presented in the supplementary materials.

### Participant Demographics

Demographics for all participants and by intervention are presented in Table 2. The majority of participants were women (76.7%), single (50.7%), and white (76.7%). The average participant lived with four people, left the house several times a week (55.3%), did not leave the house for work (56.6%), exercised outside at least several times a week (62.6%), and interacted with someone outside their household (virtually or online) several times a week (63.3%). Self-care and amount of exercise per week were significantly different by intervention, and relationship status was marginally significant by intervention. Therefore, these three variables were controlled for in further analyses. Randomisation was successful for the rest of the variables.

**Table 2:** Participants demographic information

		All		Nostalgia *n* = 40		Gratitude *n* = 36		BPS *n* = 37		Control *n* = 37		*F*	*X*^*2*^	*P*
Age (range 18 – 82 years old)	*M*(*SD)* =	26.29	12.25	27.25	14.77	25.59	11.26	25.33	11.18	27.00	11.75	0.19		.903
*Gender*													14.35	.110
Woman	*n*(%) =	115	76.7	28	70.0	25	69.4	29	78.4	33	89.2			
Man	*n*(%) =	33	22.0	12	30.0	11	30.6	8	21.6	2	6.1			
Non-Binary	*n*(%) =	1	0.7	0	0	0	0	0	0	1	2.7			
Other	*n*(%) =	1	0.7	0	0	0	0	0	0	1	2.7			
*Ethnicity*													9.56	.847
Asian	*n*(%) =	14	9.3	4	10.0	4	11.1	5	13.5	1	2.7			
Black	*n*(%) =	6	4.0	2	5.0	2	5.6	2	5.4	0	0.0			
Latina	*n*(%) =	1	0.7	1	2.5	0	0.0	0	0.0	0	0.0			
Mixed	*n*(%) =	8	5.3	2	5.0	1	2.8	2	5.4	3	8.1			
White	*n*(%) =	115	76.7	2	5.0	2	5.6	1	2.7	1	2.7			
Other	*n*(%) =	6	4.0	29	72.5	27	75.0	27	73.0	32	86.5			
*Relationship status*													15.92	.069
Single	*n*(%) =	76	50.7	22	55.0	21	58.3	16	43.2	17	45.9			
Dating one or more people	*n*(%) =	9	6.0	0	0.0	3	8.3	5	13.5	0	0.0			
In a committed relationship	*n*(%) =	39	26.0	12	30.0	7	19.4	8	21.6	12	32.4			
Engaged/married	*n*(%) =	26	17.3	6	15.0	5	13.9	8	21.6	8	21.6			
*Confirmed case of COVID-19?*													3.85	.279
Yes	*n*(%) =	7	4.7	3	7.7	0	0.0	1	2.7	3	8.1			
No	*n*(%) =	142	94.7	36	92.3	36	100.0	36	97.3	34	91.9			
*Number of people living with (range 0 – 55)*	*M*(*SD)* =	3.58	4.95	3.33	2.46	4.47	9.05	3.22	2.52	3.33	2.61	0.54		.648
*Number of times outside in last two week*										13.29	.150			
Not at all	*n*(%) =	14	9.3	1	2.5	3	8.3	4	10.8	6	16.2			
Several times a week	*n*(%) =	83	55.3	19	47.5	18	50.0	26	70.3	20	54.1			
Once a day	*n*(%) =	31	20.7	13	32.5	9	25.0	4	10.8	5	13.5			
More than once a day	*n*(%) =	22	4.7	7	17.5	6	16.7	3	8.1	6	16.2			
*Number of Times Outside in Last Two Week for Work*										12.70	.177			
Not at all	*n*(%) =	84	56.0	17	42.5	23	63.9	21	56.8	23	62.2			
Several times a week	*n*(%) =	52	34.7	15	37.5	11	30.6	13	35.1	13	35.1			
Once a day	*n*(%) =	9	6.0	4	10.0	1	2.8	3	8.1	1	2.7			
More than once a day	*n*(%) =	5	3.3	4	10.0	1	2.8	0	0.0	0	0.0			
*Number of Times Outside in Last Two Week for Exercise*										16.92	.050*			
Not at all	*n*(%) =	56	37.3	14	35.0	12	33.3	12	32.4	18	48.6			
Several times a week	*n*(%) =	65	43.3	19	47.5	14	38.9	23	62.2	9	24.3			
Once a day	*n*(%) =	23	15.3	5	12.5	8	22.2	2	5.4	8	21.6			
More than once a day	*n*(%) =	6	4.0	2	5.0	2	5.6	0	0.0	2	5.4			
*Amount of In Person or Virtual Social Interaction*										6.90	.648			
Not at all	*n*(%) =	14	9.3	4	10.0	2	5.6	5	13.5	3	8.3			
Several times a week	*n*(%) =	95	63.3	20	50.0	26	72.2	24	64.9	25	69.4			
Once a day	*n*(%) =	18	12.0	7	17.5	4	11.1	4	10.8	3	8.3			
More than once a day	*n*(%) =	22	14.7	9	22.5	4	11.1	4	10.8	5	13.9			
Self-Care behaviours (range 1.87 – 5.00)	*M*(*SD)* =	3.22	0.52	3.26	0.65	3.08	0.39	3.28	0.54	3.26	0.45	2.75		.046*

### Manipulation Checks

**Fig. 2:**
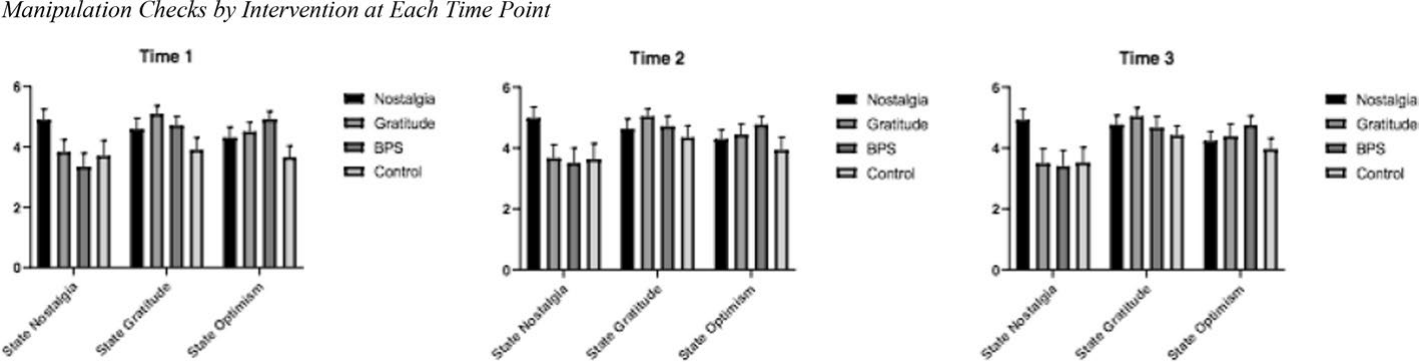
Manipulation checks by intervention at each time point

Manipulation checks were conducted for T1-T3 through one-way ANCOVAs and further examined using post hoc tests with Bonferroni correction (see Table 3 and Fig. 2).

**Table 3:** Manipulation checks T1-T4 by intervention

	Nostalgia (1) *n* = 40		Gratitude (2) *n* = 36		BPS (3) *n* = 37		Control (4) *n* = 37		Main effect of intervention			Post hoc
	*M*	SD	*M*	SD	*M*	SD	*M*	SD	*F*(3,145)	*p*	η_p_^2^	
T1 State Nostalgia	4.90	1.08	3.83	1.20	3.34	1.36	3.70	1.55	10.83	.0001	.19	1 > 2, 3, 4*** *d* = 0.89, *d* = 1.53, *d* = 0.77 2 > 3* *d* = 0.36
T1 State Gratefulness	4.59	1.12	5.09	0.81	4.71	0.92	3.90	1.21	13.20	.0001	.22	1, 2, 3 > 4** *d* = 0.57, *d* = 0.98, *d* = 0.67 2 > 1, 3** *d* = 0.45, *d* = 0.41
T1 State Optimism	4.31	1.08	4.50	0.92	4.92	0.75	3.66	1.12	11.60	.0001	.20	3 > 1* *d* = 0.56 1, 2, 3 > 4** *d* = 0.58, *d* = 0.75, *d* = 1.13
T2 State Nostalgia	4.98	1.16	3.66	1.32	3.51	1.48	3.63	1.53	10.67	.0001	.18	1 > 2, 3, 4*** *d* = 1.00, *d* = 0.99, *d* = 0.88
T2 State Gratefulness	4.63	1.01	5.03	0.79	4.71	1.03	4.34	1.19	5.80	.001	.11	2 > 1, 3, 4* *d* = 0.40, *d* = 0.31, *d* = 0.58
T2 State Optimism	4.29	0.94	4.44	1.04	4.77	0.78	3.93	1.25	5.40	.002	.10	2, 3 > 4** *d* = 0.41, *d* = 0.67
T3 State Nostalgia	4.92	1.16	3.49	1.43	3.40	1.53	3.52	1.50	10.69	.0001	.18	1 > 2, 3, 4*** *d* = 1.00, *d* = 1.00, *d* = 0.93
T3 State Gratefulness	4.77	0.95	5.03	0.86	4.66	1.11	4.41	0.92	4.63	.004	.09	2 > 3, 4* *d* = 0.33, *d* = 0.67
T3 State Optimism	4.23	0.95	4.39	1.17	4.75	0.92	3.96	1.07	4.47	.005	.09	3 > 1, 4* *d* = 0.57, *d* = 0.74

^*^p < 0.05, **p < 0.01, ***p < 0.001

The results showed that participants in the nostalgia intervention had higher state nostalgia at T1 (*p* < 0.001, *d* = 0.77), T2 (*p* < 0.001, *d* = 0.88), and T3 (*p* < 0.001, *d* = 0.93). Additionally, participants in the gratitude intervention had higher state gratitude at T1 (*p* < 0.001, *d* = 0.98), T2 (*p* < 0.001, *d* = 0.58), and T3 (*p* < 0.001, *d* = 0.67). Last, participants in the BPS intervention had higher state optimism than those in the control at T1 (*p* < 0.001, *d* = 1.13), T2 (*p* < 0.01, *d* = 0.67), and T3 (*p* < 0.01, *d* = 0.74). This shows that the interventions were effective at inducing nostalgia, gratitude, or optimism.

### Intervention Differences at Each Time Point

To examine the effect of intervention at each point, a series of ANCOVAs were conducted to assess a difference by intervention on social connectedness, self-esteem, optimism, happiness, and fear of COVID-19; see Table 4 for means and standard deviations.

**Table 4:** Well-being and fear of COVID over time by intervention (Means, SDs)

	Nostalgia (1) *n* = 40				Gratitude (2) *n* = 36				BPS (3) *n* = 37				Control (4) *n* = 37			
	T1	T2	T3	T4	T1	T2	T3	T4	T1	T2	T3	T4	T1	T2	T3	T4
Social Connectedness	4.77 (0.81)	4.46 (1.18)	4.54 (1.10)	4.63 (0.99)	4.86 (1.01)	4.83 (0.98)	4.60 (1.00)	4.68 (1.09)	4.93 (0.81)	4.89 (0.87)	4.75 (1.20)	4.88 (1.04)	4.50 (1.00)	4.68 (0.99)	4.66 (1.03)	4.68 (0.89)
Self-Esteem	4.30 (1.21)	4.15 (1.20)	4.31 (1.22)	4.25 (1.33)	4.24 (1.29)	4.22 (1.28)	4.31 (1.17)	4.38 (1.10)	4.43 (0.91)	4.53 (0.81)	4.51 (1.04)	4.49 (0.93)	3.83 (1.33)	4.03 (1.31)	4.14 (1.21)	4.14 (1.24)
Optimism	4.34 (1.12)	4.25 (1.27)	4.36 (1.17)	4.19 (1.29)	4.35 (1.11)	4.24 (1.19)	4.17 (1.26)	4.29 (1.14)	4.68 (0.83)	4.70 (0.74)	4.57 (1.16)	4.60 (0.98)	4.11 (1.02)	4.14 (1.15)	4.26 (1.04)	4.18 (0.98)
Meaning	4.91 (1.01)	4.71 (1.08)	4.81 (1.15)	4.75 (1.18)	4.71 (1.10)	4.58 (1.18)	4.56 (1.27)	4.56 (1.22)	4.88 (0.78)	4.80 (0.80)	4.51 (1.16)	4.68 (0.89)	4.43 (1.14)	4.61 (1.22)	4.61 (1.05)	4.33 (1.20)
Happiness	4.35 (1.28)	4.47 (1.29)	4.53 (1.20)	4.61 (1.29)	4.45 (1.29)	4.35 (1.29)	4.38 (1.30)	4.22 (1.31)	4.49 (1.27)	4.59 (1.26)	4.57 (1.19)	4.63 (1.20)	4.26 (1.36)	4.39 (1.26)	4.57 (1.09)	4.31 (1.33)
Fear of COVID-19	1.92 (0.76)	1.87 (0.76)	1.76 (0.72)	1.71 (0.78)	2.07 (0.65)	2.02 (0.81)	1.83 (0.90)	1.83 (0.90)	2.34 (0.78)	2.02 (0.83)	1.98 (0.86)	1.86 (0.81)	2.49 (0.96)	2.40 (0.94)	2.36 (1.08)	2.30 (1.04)

The results showed that at T1, there was a main effect of the intervention on social connectedness, self-esteem, and fear of COVID-19; see Table 5. Planned contrasts showed that participants in the gratitude (*p* < 0.05, *d* = 0.36) and BPS (*p* < 0.05, *d* = 0.43) intervention reported significantly higher social connectedness than participants in the control intervention, to a small effect size. For self-esteem, participants in the nostalgia (*p* < 0.05, *d* = 0.35), gratitude (*p* < 0.05, *d* = 0.30), and BPS (*p* < 0.05, *d* = 0.45) intervention showed significantly higher self-esteem than participants in the control intervention, all to a small effect size. In terms of fear of COVID-19, participants in the nostalgia intervention showed significantly less fear of COVID-19 than participants in the BPS (*p* < 0.05, *d* = 0.54) and control (*p* < 0.05, *d* = 0.60) intervention, to a medium effect size. Additionally, participants in the gratitude intervention showed significantly less fear of COVID-19 than participants in the control intervention (*p* < 0.05, *d* = 0.45), to small effect. This indicates that the nostalgia, gratitude, and BPS interventions show immediate increases in self-esteem; gratitude and BPS interventions show immediate increases in social connectedness; last, the nostalgia and gratitude intervention indicate an immediate reduction in fear of COVID-19.

**Table 5:** Well-Being and Fear of COVID-19 by Intervention at each time point (*F, p, η*_p_^2^ and and Post hocs)

	Main effect of intervention			Post hoc
	*F*(3,145)	*p*	*η*_p_^2^	
T1 Social Connectedness	3.34	.021	.07	2, 3 > 4* *d* = 0.36, *d* = 0.43
T1 Self-Esteem	2.80	.041	.06	1, 2, 3 > 4* *d* = 0.35, *d* = 0.30, *d* = 0.45
T1 Optimism	2.52	.061	.05	
T1 Meaning	2.52	.060	.05	
T1 Happiness	0.98	.406	.02	
T1 Fear of COVID-19	4.42	.005	.09	4 > 1, 2* *d* = 0.60, *d* = 0.45 3 > 1* *d* = 0.54
T2 Social Connectedness	2.70	.048	.05	2 > 1** *d* = 0.31
T2 Self-Esteem	1.48	.224	.03	
T2 Optimism	1.98	.119	.04	
T2 Meaning	0.23	.878	.01	
T2 Happiness	0.25	.861	.01	
T2 Fear of COVID-19	2.92	.068	.06	4 > 1* *d* = 0.56
T3 Social Connectedness	0.45	.715	.01	
T3 Self-Esteem	0.89	.447	.02	
T3 Optimism	0.40	.751	.01	
T3 Meaning	0.80	.494	.02	
T3 Happiness	0.06	.979	.00	
T3 Fear of COVID-19	3.69	.013	.07	4 > 1, 2** *d* = 0.55, *d* = 0.49
T4 Social Connectedness	0.76	.521	.02	
T4 Self-Esteem	1.50	.218	.03	
T4 Optimism	1.53	.210	.03	
T4 Meaning	1.76	.158	.04	
T4 Happiness	0.62	.603	.01	
T4 Fear of COVID-19	3.64	.014	.07	4 > 1, 2, 3* *d* = 0.57, *d* = 0.44, *d* = 0.42

1 = nostalgia, 2 = gratitude, 3 = BPS, 4 = control. *p < 0.05, **p < 0.01, ***p < 0.001

At T2, the results showed a main effect of the intervention on social connectedness and fear of COVID-19, see Table 5. Planned contrasts showed that participants in the gratitude intervention showed significantly more social connectedness than participants in the nostalgia intervention, to a small effect size (*p* < 0.01, *d* = 0.31). For fear of COVID-19, participants in the nostalgia intervention showed significantly less fear of COVID-19 than participants in the control intervention, to a medium effect size (*p* < 0.05, *d* = 0.56). This indicates that after two interventions, gratitude is effective at increasing social connectedness compared to nostalgia, but nostalgia is effective at reducing COVID-19 compared to the control intervention.

There was a main effect of the intervention on fear of COVID-19 at T3, see Table 5. Planned contrasts show that participants in the nostalgia (*p* < 0.01, *d* = 0.55) and gratitude (*p* < 0.01, *d* = 0.49) intervention showed significantly less fear of COVID-19 than participants in the control intervention, to a medium effect. This indicates that after three interventions, nostalgia and gratitude interventions are effective at reducing fear of COVID-19 compared to the control intervention.

The results showed a main effect of the intervention on fear of COVID-19 at T4, see Table 5. Planned contrasts revealed that participants in the nostalgia (*p* < 0.05, *d* = 0.57), gratitude (*p* < 0.05, *d* = 0.44), and BPS (*p* < 0.05, *d* = 0.42) intervention showed significantly less fear of COVID-19 compared to participants in the control intervention, to small and medium effect sizes. This indicates that at one-week post-intervention, all PPIs effectively reduce fear of COVID-19 compared to the control intervention.

### The Impact of the Interventions Over Time

**Fig. 3:**
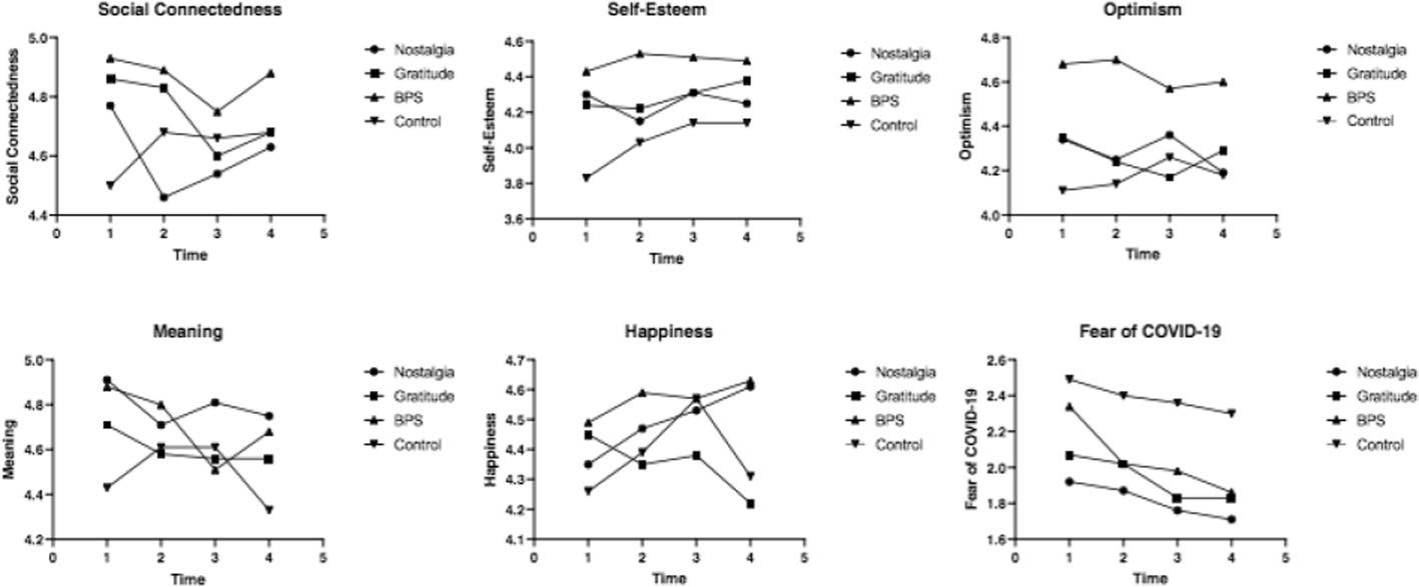
Well-being and fear of COVID-19 by intervention and over time

The effect of the intervention over time on changes in social connectedness, self-esteem, optimism, happiness, and fear of COVID-19 were assessed with a 4 (time: T1, T2, T3, T4) × 4 (intervention: nostalgia, gratitude, BPS, control) mixed ANCOVA, see Table 6 and Fig. 3. The results showed that the interaction between time and intervention was non-significant for all variables: social connectedness, self-esteem, optimism, happiness, and fear COVID-19. This is not surprising given that T1 was not a baseline measurement and suggests that after the first intervention, repeated use sustains well-being rather than continuing to increase well-being. However, the effect of time was significant for self-esteem (*p* < 0.05, η_p_^2^ = 0.06).

**Table 6:** Well-being and fear of COVID-19 by time and intervention (F, p, *η*_p_^2^)

	Main effect of time			Interaction effect of Time × Intervention		
	*F*(3,145)	*p*	*η*_p_^2^	*F*(3,145)	*p*	*η*_p_^2^
Social Connectedness	0.01	.999	.00	1.18	.304	.02
Self-Esteem	3.00	.033	.06	0.79	.625	.02
Optimism	0.21	.892	.00	0.66	.749	.01
Meaning	2.10	.103	.04	1.32	.222	.03
Happiness	0.56	.640	.01	1.82	.062	.04
Fear of COVID-19	0.29	.833	.01	1.60	.113	.03

## Discussion

The COVID-19 pandemic has had negative effects on well-being. Previous research (Dennis et al., 2020) has compared the effectiveness of three PPIs on well-being during COVID-19. The results showed that gratitude and optimism might hold more benefits on well-being than nostalgia or the control. In this study, we sought to replicate the findings by Dennis et al. (2020) and extend the interventions over a two-week period. This study aimed to compare the effects of three PPIs at each time point (T1-T4) on well-being related constructs during the UK’s second lockdown and then compare the PPIs' effectiveness on well-being related constructs across the two weeks and follow-up.

### Immediate Effects

After the first intervention, there were differences by intervention on social connectedness, self-esteem, and fear of COVID-19. In terms of social connectedness, the results showed that after the first intervention, individuals who underwent the gratitude or BPS intervention reported more social connectedness than individuals in the control intervention, both to a small effect. The findings are in line with Dennis et al. (2020) that gratitude and BPS immediately led to more increases in social connectedness.

In terms of self-esteem, immediately after the first intervention, participants in the nostalgia, gratitude, and BPS intervention showed increased self-esteem compared to participants in the control intervention. Nostalgia, gratitude, and BPS interventions have previously been shown to increase self-esteem (Cheung et al., 2013; Lin, 2015; Owens & Patterson, 2013; Rash et al., 2011). However, it is in contrast with Dennis et al. (2020), where no differences were observed in self-esteem, although the effect was small and did not last beyond the first intervention.

The results also indicated that after the first intervention, individuals who had undergone the nostalgia and gratitude interventions reported less fear of COVID-19 than individuals in the BPS and control interventions. Fear of COVID-19 was not explored in Dennis et al. (2020), but it does extend the findings to show the further benefits of PPI during COVID-19.

The results suggest that nostalgia and gratitude have an immediate buffering effect on the threat of COVID-19. This supports research showing nostalgia can buffer negative psychological states and maintain psychological homeostasis (Sedikides et al., 2015b), and gratitude can mitigate health anxiety (Otto et al., 2016).

### Longer Term Effects

This study also showed extended effects of the interventions showing differences in social connectedness and fear of COVID-19. After the second intervention, individuals in the gratitude intervention showed higher social connectedness than those in the nostalgia intervention, with no differences in the BPS intervention. There were no further differences by intervention on social connectedness, showing that gratitude and BPS have immediate effects at increasing social connectedness (compared to nostalgia and control). However, this ability to increase social connectedness reduces over time. Therefore, gratitude and BPS may be better at immediately increasing social connectedness than nostalgia and control. This may be because people often express gratitude for close others (Emmons et al., 2003), with family being a central component of gratitude (Lambert et al., 2009). Gratitude then works through directing attention towards the positives and savouring those positives (Lau & Cheng, 2013; Seligman et al., 2006). Additionally, BPS narratives heavily include family and partners that increase positive mood (Carrillo et al., 2019b). In the case of social connectedness, gratitude may remind people of the social connections they do have, and BPS may increase social connections through thoughts of family and partners. Then nostalgia may be less effective compared to gratitude and BPS. Although a common feature is commonly is other people and close relationships (Abeyta et al., 2015; Wildschut et al., 2006) and when being nostalgic, it is common to compare the past to the present (Davalos et al., 2015). Thus, when the present involves lockdown restrictions, social distancing, and a lack of social interaction, it may create a sense of loss, particularly when thinking about interacting with others.

The results also indicated longer term benefits on reducing fear of COVID-19. After the second intervention (T2), the nostalgia intervention reduced fear of COVID-19 compared to the control. Following the third intervention (T3), nostalgia and gratitude interventions were more effective at reducing fear of COVID-19 compared to the control. Then at the follow-up (T4), all interventions were more effective at reducing fear of COVID-19 than the control. At follow-up, nostalgia showed the biggest effect at reducing fear of COVID-19, compared to the control, followed by gratitude, then BPS. The results show that nostalgia and gratitude have immediate and sustained effects at reducing COVID-19, whereas BPS reduced fear of COVID-19 through repeated engagement.

The results suggest that nostalgia is most beneficial at reducing fear of COVID-19 and that gratitude and BPS can also reduce fear of COVID-19 compared to the control. Previous research has shown that the nostalgia intervention increases health optimism (Kersten et al., 2016), reducing health risk perception (Ferrer & Klein, 2015; Radcliffe & Klein, 2002). Moreover, recalling nostalgic memories increase perceptions of youthfulness, vitality, and health confidence (Abeyta & Routledge, 2016). As well as promoting health optimism, participants were instructed to think of nostalgic memories from before the COVID-19 pandemic; recalling memories from before the pandemic that does not include COVID-19 may help reduce the fear of COVID-19. In terms of gratitude, a recent meta-analysis showed that gratitude had a small effect on reducing the symptoms of anxiety at post-intervention and follow-up (Cregg & Cheavans, 2021), similar to the current results. Gratitude has been suggested to reduce anxiety and specific anxiety (e.g., death anxiety) by directing attention towards positivity and increasing self-reassurance (Lau & Cheng, 2013; Petrocchi & Couyoumdjian, 2016). Although BPS has previously been shown to increase positive expectancies, reduce negative expectancies, and reduce worrying (Meevissen et al., 2011; Nicolson et al., 2020), the effects of BPS on reducing fear of COVID-19 were only noticeable at follow-up. In sum, the results suggest that nostalgia and gratitude have immediate effects on reducing fear of COVID-19, with nostalgia showing stronger effects at reducing fear of COVID-19. In contrast, BPS takes repeated engagement before a reduction in fear of COVID-19 is observed.

### Effects Over Time

In terms of the second aim, the results showed no difference by intervention across time on well-being. However, previous research has compared intervention effectiveness across time using baselines scores and comparing that to post-intervention and follow-up scores (e.g., Meevissen et al., 2011; Peters et al., 2010; Seligman et al., 2005). In the current study, baseline scores were not assessed due to time constraints of the lockdown restrictions, and T1 was immediately after the first intervention. Therefore, the results show no intervention differences from repeated engagement on well-being.

### Practical Implications

The current research has implications for future lockdowns and quarantines. Engaging in PPIs may improve well-being, and these interventions can be chosen depending on the most necessary aspects of well-being. Additionally, the current study also has implications for understanding PPIs, in that from this study combined with Dennis et al. (2020), the results indicate that PPIs have different benefits. For example, gratitude and BPS seem to be best at buffering social isolation and nostalgia for buffering fear of COVID-19. Therefore, the same approach cannot be taken for combating all psychological distress. Loneliness may be best buffered by gratitude and BPS whereas, nostalgia may best buffer health anxiety.

### Limitations

The data from this study allowed us to assess the effect of repeated engagement with three PPIs on well-being. There are some limitations that need to be considered. First, baseline scores were not measured. Therefore, we are unable to account for individual variation in well-being prior to the PPIs. Additionally, the absence of a baseline means we are unable to draw conclusions on to what degree the PPIs increased well-being from before the intervention and are only able to compare the effects of PPIs. Additionally, all participants were from the UK, and most were in their mid-twenties, single, and students, yet the COVID-19 pandemic has affected all populations worldwide. Therefore, the results may not be generalisable for all populations. Third, on the whole, the effect sizes of the intervention were small. However, participants did only engage in three rounds of the intervention and longer engagement (e.g., two months) may show larger increases in well-being (Bolier et al., 2013). Fourth, the gratitude and optimism manipulation checks are not validated measures, therefore, we do not know how well it measures state gratitude and state optimism. Last, we could not control the amount participants engaged in the PPI. Last, although participants had to stay on the intervention page for two minutes this does not mean they engaged with it for two minutes and differences in engagement of PPIs may lead to varying increases in well-being gained. Participants may not have engaged in the time-orientation they were assigned to, for example, although asked to think about something they were grateful for today, participants could have thought about something they were grateful for from a previous day.

## Conclusions

To conclude, our findings suggest that the PPIs of nostalgia, gratitude, and BPS are all effective at increasing and maintaining aspects of well-being; however, the PPIs vary in their benefits. Nostalgia showed the largest effect at reducing fear of COVID-19, whereas gratitude and BPS are more effective at increasing social connectedness. Therefore, a targeted approach is recommended; for example, if an individual has health anxiety over the pandemic nostalgia may be best suited, however, if an individual is feeling lonely because of reduced social contact may best benefit from gratitude or BPS. In sum, our results demonstrate the effectiveness of repeated engagement with nostalgia, gratitude, and BPS interventions on well-being during lockdown. Furthermore, implementing these interventions in weekly practises can aid well-being and buffer against mental illness during the COVID-19 pandemic as well as when lockdown restrictions ease.

## Supplementary Information

Below is the link to the electronic supplementary material.

Supplementary file1 (DOCX 28 KB)

## Data Availability

The data that supports the findings of this study are available from the corresponding author, AD, upon reasonable request.
