# Supplementary material for: Nostalgia, Gratitude, or Optimism: The Impact of a Two-Week Intervention on Well-Being During COVID-19
Source: J Happiness Stud. 2022 Mar 19;23(6):2613–34. doi: 10.1007/s10902-022-00513-6 (PMC8934022; doi:10.1007/s10902-022-00513-6)
Supplement: Supplementary file 1 — Supplementary file1 (DOCX 28 KB) [file 10902_2022_513_MOESM1_ESM.docx]

**Supplementary Materials**

Key for Correlation Matrix

| **Number in correlation matric below** | **Variable** |
| --- | --- |
| 1 | T1 social connectedness |
| 2 | T1Vmeaning |
| 3 | T1 self-esteem |
| 4 | T1 optimism |
| 5 | T1 happiness |
| 6 | T1 fear of COVID-19 |
| 7 | T2 social connectedness |
| 8 | T2 meaning |
| 9 | T2 self-esteem |
| 10 | T2 optimism |
| 11 | T2 happiness |
| 12 | T2 fear of COVID-19 |
| 13 | T3 social connectedness |
| 14 | T3 meaning |
| 15 | T3 self-esteem |
| 16 | T3 optimism |
| 17 | T3 happiness |
| 18 | T3 fear of COVID-19 |
| 19 | T4 social connectedness |
| 20 | T4 meaning |
| 21 | T4 self-esteem |
| 22 | T4 optimism |
| 23 | T4 happiness |
| 24 | T4 fear of COVID-19 |

Correlation Matrix between outcome variables

|  | 1 | 2 | 3 | 4 | 5 | 6 | 7 | 8 | 9 | 10 | 11 | 12 | 13 | 14 | 15 | 16 | 17 | 18 | 19 | 20 | 21 | 22 | 23 | 24 |
| --- | --- | --- | --- | --- | --- | --- | --- | --- | --- | --- | --- | --- | --- | --- | --- | --- | --- | --- | --- | --- | --- | --- | --- | --- |
| 1 | - | .662** | .544** | .570** | .483** | -0.024 | .650** | .459** | .438** | .447** | .467** | -0.082 | .580** | .499** | .418** | .452** | .456** | -0.150 | .598** | .554** | .434** | .448** | .460** | -0.112 |
| 2 | .662** | - | .691** | .688** | .496** | -0.097 | .472** | .703** | .533** | .512** | .477** | -0.133 | .408** | .624** | .481** | .485** | .430** | -.201* | .466** | .723** | .522** | .548** | .465** | -0.159 |
| 3 | .544** | .691** | - | .699** | .609** | -0.137 | .474** | .606** | .735** | .607** | .633** | -.187* | .451** | .562** | .701** | .532** | .591** | -.200* | .439** | .601** | .651** | .594** | .597** | -0.145 |
| 4 | .570** | .688** | .699** | - | .556** | -0.103 | .472** | .552** | .551** | .673** | .490** | -.179* | .477** | .567** | .525** | .598** | .477** | -.180* | .442** | .547** | .515** | .639** | .467** | -.180* |
| 5 | .483** | .496** | .609** | .556** | - | -.176* | .473** | .447** | .558** | .555** | .823** | -.178* | .492** | .483** | .536** | .473** | .814** | -.183* | .427** | .437** | .511** | .488** | .806** | -.163* |
| 6 | -0.024 | -0.097 | -0.137 | -0.103 | -.176* | - | -0.003 | -0.113 | -0.157 | -0.110 | -.195* | .880** | -.195* | -.237** | -.212** | -.178* | -.187* | .849** | -0.083 | -0.105 | -0.152 | -0.126 | -.196* | .825** |
| 7 | .650** | .472** | .474** | .472** | .473** | -0.003 | - | .725** | .620** | .625** | .601** | -0.060 | .620** | .559** | .527** | .516** | .534** | -0.034 | .725** | .636** | .548** | .562** | .526** | -0.035 |
| 8 | .459** | .703** | .606** | .552** | .447** | -0.113 | .725** | - | .707** | .722** | .612** | -.161* | .530** | .712** | .610** | .598** | .484** | -0.127 | .638** | .776** | .648** | .665** | .551** | -0.108 |
| 9 | .438** | .533** | .735** | .551** | .558** | -0.157 | .620** | .707** | - | .783** | .704** | -.238** | .440** | .549** | .719** | .538** | .580** | -.179* | .521** | .595** | .746** | .651** | .621** | -0.145 |
| 10 | .447** | .512** | .607** | .673** | .555** | -0.110 | .625** | .722** | .783** | - | .632** | -.175* | .539** | .619** | .645** | .687** | .545** | -0.126 | .514** | .574** | .669** | .752** | .590** | -0.104 |
| 11 | .467** | .477** | .633** | .490** | .823** | -.195* | .601** | .612** | .704** | .632** | - | -.225** | .542** | .535** | .641** | .538** | .867** | -.199* | .560** | .551** | .647** | .561** | .906** | -.163* |
| 12 | -0.082 | -0.133 | -.187* | -.179* | -.178* | .880** | -0.060 | -.161* | -.238** | -.175* | -.225** | - | -.255** | -.280** | -.283** | -.242** | -.219** | .894** | -0.140 | -.164* | -.206* | -.177* | -.222** | .893** |
| 13 | .580** | .408** | .451** | .477** | .492** | -.195* | .620** | .530** | .440** | .539** | .542** | -.255** | - | .753** | .716** | .724** | .586** | -.237** | .691** | .540** | .561** | .592** | .537** | -.257** |
| 14 | .499** | .624** | .562** | .567** | .483** | -.237** | .559** | .712** | .549** | .619** | .535** | -.280** | .753** | - | .745** | .791** | .562** | -.231** | .625** | .765** | .611** | .676** | .555** | -.235** |
| 15 | .418** | .481** | .701** | .525** | .536** | -.212** | .527** | .610** | .719** | .645** | .641** | -.283** | .716** | .745** | - | .760** | .638** | -.257** | .605** | .650** | .788** | .737** | .640** | -.244** |
| 16 | .452** | .485** | .532** | .598** | .473** | -.178* | .516** | .598** | .538** | .687** | .538** | -.242** | .724** | .791** | .760** | - | .591** | -.202* | .587** | .641** | .614** | .779** | .578** | -.240** |
| 17 | .456** | .430** | .591** | .477** | .814** | -.187* | .534** | .484** | .580** | .545** | .867** | -.219** | .586** | .562** | .638** | .591** | - | -.205* | .542** | .502** | .578** | .568** | .894** | -.191* |
| 18 | -0.150 | -.201* | -.200* | -.180* | -.183* | .849** | -0.034 | -0.127 | -.179* | -0.126 | -.199* | .894** | -.237** | -.231** | -.257** | -.202* | -.205* | - | -0.150 | -0.145 | -.196* | -.165* | -.211** | .910** |
| 19 | .598** | .466** | .439** | .442** | .427** | -0.083 | .725** | .638** | .521** | .514** | .560** | -0.140 | .691** | .625** | .605** | .587** | .542** | -0.150 | - | .745** | .688** | .689** | .569** | -0.149 |
| 20 | .554** | .723** | .601** | .547** | .437** | -0.105 | .636** | .776** | .595** | .574** | .551** | -.164* | .540** | .765** | .650** | .641** | .502** | -0.145 | .745** | - | .731** | .730** | .580** | -0.118 |
| 21 | .434** | .522** | .651** | .515** | .511** | -0.152 | .548** | .648** | .746** | .669** | .647** | -.206* | .561** | .611** | .788** | .614** | .578** | -.196* | .688** | .731** | - | .806** | .631** | -.168* |
| 22 | .448** | .548** | .594** | .639** | .488** | -0.126 | .562** | .665** | .651** | .752** | .561** | -.177* | .592** | .676** | .737** | .779** | .568** | -.165* | .689** | .730** | .806** | - | .614** | -.160* |
| 23 | .460** | .465** | .597** | .467** | .806** | -.196* | .526** | .551** | .621** | .590** | .906** | -.222** | .537** | .555** | .640** | .578** | .894** | -.211** | .569** | .580** | .631** | .614** | - | -.186* |
| 24 | -0.112 | -0.159 | -0.145 | -.180* | -.163* | .825** | -0.035 | -0.108 | -0.145 | -0.104 | -.163* | .893** | -.257** | -.235** | -.244** | -.240** | -.191* | .910** | -0.149 | -0.118 | -.168* | -.160* | -.186* | - |
